# Antilisterial Activity of Polypropylene Film Coated with Chitosan with Propolis and/or Bee Pollen in Food Models

**DOI:** 10.1155/2019/7817063

**Published:** 2019-02-20

**Authors:** Krzysztof Skowron, Joanna Kwiecińska-Piróg, Katarzyna Grudlewska, Grzegorz Gryń, Natalia Wiktorczyk, Maciej Balcerek, Daniel Załuski, Ewa Wałecka-Zacharska, Stefan Kruszewski, Eugenia Gospodarek-Komkowska

**Affiliations:** ^1^Department of Microbiology, Nicolaus Copernicus University in Toruń, Collegium Medicum of L. Rydygier in Bydgoszcz, Poland; ^2^Plant Breeding and Acclimatization Institute – National Research Institute, Al. Powstańców Wlkp. 10, 85-090 Bydgoszcz, Poland; ^3^Department of Pharmacognosy, Ludwik Rydygier Collegium Medicum, Nicolaus Copernicus University, 9 Marie Curie-Skłodowska Street, 85-094 Bydgoszcz, Poland; ^4^Department of Food Hygiene and Consumer Health, Wrocław University of Environmental and Life Sciences, 31 C.K. Norwida St., 50-375 Wrocław, Poland; ^5^Biophysics Department, Faculty of Pharmacy, Collegium Medicum of Nicolaus Copernicus University, Jagiellońska 13-15 St., 85–067 Bydgoszcz, Poland

## Abstract

The aim of the study was to evaluate the effect of propylene film coated with solution of chitosan (CH), ethanolic extracts of propolis (EEP), and bee pollen (EEBP) and its combination on* L. monocytogenes *number in wrapped salmon, salami, and cheese. Sterile fragments of propylene film were coated with solution containing CH, CH+EEP, CH+EEBP, and CH+EEP+EEBP. The coated film was applied directly after preparation (AP) after 10 days of storage from preparation (AS).* L. monocytogenes* strains isolated from cheese, salmon, and salami were transferred on adequate food type. ATCC 19111 reference strain was placed on all examined slices. Contaminated slices were wrapped in the coated film. The film adhered strictly to the slices surface and was left for 0, 1, 6, 12, and 24 hours. Antilisterial activity of AP film was additionally assessed during 15-day storage of products wrapped in the coated film. In conclusion, the chitosan-coated film exhibited antibacterial activity. Incorporation of EPP and EEBP enhanced this activity. The antilisterial activity depended on the type and concentration of solutions, the types of food, and the origin of strains. This study proved that the time that passed since the use of coated film for packing food was of great importance.

## 1. Introduction

The contamination of food products after proper processing (e.g., temperature processing) is a severe problem. The bacteria can be transmitted from the environment to food products during cutting, portioning, weighing, packaging, etc. [1]. More than 3,079 cases of foodborne infections were reported in Europe in 2017 [1]. Most of these cases were caused by* Campylobacter *spp. and* Salmonella *spp. [1]. However,* Listeria monocytogenes* is also an important foodborne pathogen. The number of listeriosis cases is progressively increasing. From 2009 to 2017 an increase of 48% in the number of listeriosis cases has been observed. In 2017, 28 European countries reported 2,480 cases of invasive listeriosis, including 225 cases with fatal outcome, and the incidence rate was 0.48 cases per 100,000 people [1]. The listeriosis-related mortality rate is very high and accounts for 10-50% [2]. Neonatal infections are often severe, with a mortality rate of 30-70% [2]. The highest mortality rate is found in the central nervous system infections [3]. One of the possible solutions of this problem may be using antimicrobial packaging material that can decrease the total bacteria number in a ready-to-eat products [4].

One of the substances used in the preparation of antimicrobial packaging material is chitosan. Chitosan is produced by deacetylation of chitin being the second (after cellulose) most abundant polysaccharide found in the natural environment [5, 6]. Chitosan may be applied as a food preservative, and as a component of antimicrobial packaging material [7]. Chitosan has not been officially proclaimed GRAS by the FDA, although it has approved chitosan for medical uses such as bandages and drug encapsulation. However, one Norwegian company (Primex Ingredients ASA), which manufactures shrimp-derived chitosan, has announced in 2001 that its purified chitosan product (ChitoClear®) has achieved a GRAS self-affirmed status in the US market [8]. Its antimicrobial activity may be explained by promoting changes in cell permeability. The interactions between the amino group of chitosan and the electronegative charge on the microbial cell surface lead to leakage of the intracellular proteins and electrolytes [7]. High concentrations of chitosan may be responsible for membrane permeabilization and, in consequence, may lead to cell death [7].

Chitosan may be combined with other antimicrobial substances, e.g., propolis or bee pollen. Propolis is a resinous substance produced by bees (*Apis mellifera)* from substances collected from various vegetable sources [9]. The color, smell, and chemical composition of propolis may show a high variability related to the origin [10]. More than 300 different chemicals are found in the composition of propolis, including cinnamic and benzoic acid derivatives and flavonoids (galangin, chrysin, pinocembrin) [11, 12]. Moreover, polyphenols have been strongly associated with the antimicrobial properties of monofloral honeys [13]. In many studies, effectiveness of propolis on Gram-positive bacteria, Gram-negative bacteria, and fungal growth was observed [14–17].

Bee pollen may be also used as a compound of the antimicrobial packaging system. It consists of carbohydrates, cellulose, protein, lipids, as well as vitamins, carotenoids and phenolic compounds, sterols and terpenes. This product is the great source of nutrition. It has antibiotic, antioxidative, anti-inflammatory, antiprostatitis, antihepatotoxic, and antianemic properties and may contribute to detoxication [18]. The antimicrobial properties of bee pollen (against both Gram-positive and Gram-negative bacteria) result from the presence of flavonoids and phenolic acids. Flavonoids interfere with the metabolism of bacteria. The mechanism is based on the formation of complexes with bacterial cell walls with surface-exposed adhesins and polypeptides and/or cell membrane enzymes, which leads to disruption of cell wall integrity, blocking of ion channels, and inhibition of electron flow in the electron transport chain that determines the synthesis of adenosine triphosphate (ATP), by capturing electrons [19].

The aim of the study was to evaluate the effect of propylene film coated with solution of chitosan, ethanolic extract of propolis, and ethanolic extract of bee pollen and its combination on* L. monocytogenes *number in wrapped salmon, salami, and cheese. The impact of the storage time from film coating on antilisterial activity of film was also assessed. The purpose of the study was also to evaluate the effect of coated film on the survival and proliferation of* L. monocytogenes* during storage of packaged food.

## 2. Material and Methods

### 2.1. Material

The* Listeria monocytogenes *wild strains used in this study were isolated by authors from food products. In total there were 7 strains; 2 were isolated from smoked salmon, 2 from salami, and 2 from blue cheese, and 1 was the reference strain of* L. monocytogenes *ATCC 19111.

### 2.2. Preparation of Ethanolic Extract of Propolis and Ethanolic Extract of Bee Pollen

The procedure described by Dziedzic et al. (2013) [20] with modifications was used to prepare an ethanolic extract of propolis and ethanolic extract bee pollen. Crushed propolis (40g) or bee pollen (40 g) (apiary, Poland) was mixed with 96.0% ethanol (100ml). The suspensions were stored in the darkness at room temperature (25°C) with shaking (200 rpm, 6 hours per day) for 4 days. Then the infusion was placed at -20°C for precipitating alcohol insoluble compounds of propolis. After 24 hours, the resulting infusion was filtrated through sterile filters (0.45 *μ*m, Millipore). The 40.0% EEP and 40.0% EEBP were stored at room temperature (25°C).

### 2.3. Total Phenolic Content and Phytochemical Analysis of Ethanolic Extracts of Propolis and Bee Pollen

Total phenolic content in EEP and EEBP was determined by the Folin–Ciocalteu assay as previously described [21]. 15 *μ*l of each extract was mixed with 10-ml ultrapure water (Sigma-Aldrich), 1-ml Folin–Ciocalteu reagent (Sigma-Aldrich), and 2 ml of a 20% sodium carbonate solution (w/v) (Avantor). Then the ultrapure water (Sigma-Aldrich) was added to fill up the volume to 50 ml. After 1 h of reaction at room temperature (25°C) in the dark, the absorbance was measured at 760 nm with Beckman Spectrophotometer DU 60. Gallic acid (Sigma-Aldrich) was used as the standard for a calibration curve, and results were expressed as gallic acid equivalents (mg GAE/1 g of sample). The standard calibration was made for 1, 2, 4, 6, 8, 10, 12, and 15 *μ*g GAE/ml, in triplicate for each concentration.

Standard solution (chlorogenic acid, p-coumaric acid, 2-hydroxycinnamic acid, ferulic acid, gallic acid, caffeic acid, p-coumaric acid, syringic acid, vanillic acid, salicylic acid, and sinapic acid (50 mg each)) was dissolved in 10.0 ml of ethanol. Thin Layer Chromatography (TLC) was performed on 10×20 cm TLC sheets coated with a silica gel 60 F254 (Merck, Germany). Extracts and standards were carried out using the mobile phase: hexane: toluene: ethyl acetate: formic acid (2: 5: 2.5: 0.5 v / v / v / v) (POCH, Poland). After application of the extract and standard solution (5 *μ*l), the sheets were developed in glass chambers previously allowed to equilibrate for at least 30 min. Individual spots were placed on a TLC plate under ultraviolet light (254 nm and 366 nm) and visualized after spraying with diazotized sulfanilic acid. The Rf value of the various spots observed was calculated.

### 2.4. Preparation of Coating Solution

Coating solution based on chitosan was prepared according to the study by Torlak and Sert [22]. Chitosan (Mw 50 kDa, >90% deacetylation, Pol-Aura) was dissolved in 1.0% (v/v) water solution of acetic acid (Avantor). The final concentration of chitosan was 2.0% (w/v). The solution was supplemented with glycerol (Avantor) (final concentration 2.0% (w/v)) as a plasticizer, and Tween 20 (Sigma-Aldrich) (final concentration 0.05% (v/v)) was used to increase the wetting and adhesive properties of the coating solution.

The 40% EEP or 40% EEBP was added to the coating solution in a volumetric ratio of 1:1 or 1:3. Therefore, the final concentration of EEP or EEBP in the coating solution equal to 20.0% and 10.0%, respectively, was obtained. The coating solution with 40% EEP and 40% EEBP (EEP:EEBP, 1:1) was prepared obtaining the final concentration as described above. Different concentrations of the tested extracts were used to check whether the antimicrobial activity of films coated with them depends on their concentration and to what extent. The tested concentrations were determined based on previous studies and literature data.

### 2.5. Preparation of Coated Propylene Film

In this study we used the polypropylene film (Elzet) of thickness 25 *μ*m. Fragments of the examined film (100×100 mm) were sterilized by radiation hygiene. Sterile fragments were placed in plastic cuvettes (128×128×10 mm) and flooded (one side) with the coating solution containing CH, CH+EEP, CH+EEBP, and CH+EEP+EEBP. The wet layer of the coating solution had a thickness of about 1 mm (based on the scale on the side wall of the cuvette). The polypropylene films with coating solutions have been placed in a sterile laminar box (in plastic cuvettes) and let to dry at 25°C for 24 h (RH = 54%). The average final thickness of dry coating layer was 137.2±2.4 *μ*m. The thickness was measured with Single-Spot Thickness Measurements F20-EXR (Filmetrics). The coated film used in this study was applied: (a) directly after preparation (*AP*), (b) after 10-day storage from preparation (*AS*). The usage in the research of film stored after coating with a suitable solution was to check whether the film prepared by a relatively simple method retains its antibacterial properties and whether it can be prepared in advance and used for packaging food after storage.

### 2.6. The Impact of Coated Polypropylene Film on L. monocytogenes

*L. monocytogenes* strains used in the study were isolated from food (Section 2.1) according to the standard PN-EN ISO 11290-1:2017-07 [23] and were identified by using the MALDI-TOF MS method (Matrix-Assisted Laser Desorption Ionization Time of Flight, Mass Spectrometry) in accordance with the manufacturer procedure with MALDI Biotyper (Bruker). The obtained strains were frozen in brain-heart infusion broth (BHI, Merck) with the addition of 15% glycerol (Avantor) and stored at -80°C. For this research, the strains were grown on Columbia Agar with 5% Sheep Blood (Becton-Dickinson) and incubated for 24 hours at 37°C. After incubation, the strains were transferred once again on the same type of agar and incubated under the same conditions. Then for each strain bacterial suspensions in sterile PBS (Avantor) were prepared and adjusted to a turbidity equivalent to a 0.5 MacFarland standard (7.6×10^7^ CFU×ml^−1^) using densitometer Biosan DEN-1. Slices of smoked salmon, salami, and blue cheese Brie, measuring 2x2 cm with a thickness of 3 mm, were prepared. The slices were placed 1 m under Philips TUV TL-D-30W lamp that emitted UV-C radiation, and they were radiated 20 minutes per one side. After this time, suspensions of examined strains (200 *μ*l/slice) were placed on the surface of prepared sterile slices (separately one strain on one slice). On the surface of cheese slices* L. monocytogenes* strains isolated from cheese were transferred; on smoked salmon, strains isolated from smoked salmon; and on salami surface, strains isolated from salami.* L. monocytogenes *reference strain ATCC 19111 was placed on all of examined slices. Slices were left in a laminar box until they were dry.

Contaminated slices were wrapped in the coated film containing CH, CH+EEP, CH+EEBP, and CH+EEP+EEBP. The film adhered strictly to the slices surface and was left for: 0, 1, 6, 12, and 24 hours both for AP and AS. After this time, the film was removed from the examined surfaces, and slices were transferred to sterile PBS (100 ml) and sonicated 10 minutes in Ultrasonic DU-4 sonicator (operating frequency – 30 kHz, sonic power – 150 W, temperature 25°C) (Nickel-Electro). All samples were mixed by 10 minutes (400 rpm) and then 10-fold diluted. The dilutions were cultured on Agar* Listeria* Ottaviani & Agosti (ALOA, Merck). Colonies were counted after 24 hours of incubation at 37°C and checked once again after 48 hours of incubation. Results were expressed as colony forming units (CFU) per 1 cm^2^ of slice.

For AP the effect of polypropylene film on* L. monocytogenes* growth was evaluated additionally after 1, 5, 10, and 15 days of storage. Each experiment was done in triplicate. Contaminated slices wrapped with the uncoated polypropylene film were the positive controls. Slices of examined food products coating with film containing CH, CH+EEP, CH+EEBP, and CH+EEP+EEBP were the negative controls.

### 2.7. Statistical Analysis

Statistical differences between the number of* L. monocytogenes *CFU reisolated from slices at the last point of investigation (24 hours) depend on (a) type of food, (b) components of coating solution, and (c) time of film storage after coating. The mean value of the number of* L. monocytogenes *strains was calculated and Bonferroni post hoc test was used with *α*=0.05. The logarithmic reduction index was calculated in comparison to the positive control:

(1) $$\begin{array}{c} R\left[\;\log cfu\times {cm}^{-2}\;\right]=\log K\left(\;+\;\right)–\log A \end{array}$$

where

  K(+) is the number of* L. monocytogenes *CFU in the positive control,
  A is the number of* L. monocytogenes *CFU reisolated from examined samples at the last time of investigation.

The significance of the differences between obtained R-scores was verified using post hoc Tukey's test (*α*=0.05). All statistical calculations were made in STATISTICA 12 PL (StatSoft).

The same statistical analysis was made to establish the efficacy of coated film packaging on the number of* L. monocytogenes *CFU during the storage of packaged food.

## 3. Results and Discussion

### 3.1. Total Phenolic Content and Phytochemical Analysis of Ethanolic Extracts of Propolis and Bee Pollen

The determined equation of calibration curve was y=0.6432x+0.0285 with correlation coefficient equal to 0.97. The total phenolic content in the EEP sample was 147.57 ± 4.05 mg/g and in EEBP was 34.02±2.36 mg/g.

As a result of the analysis of propolis, six phenolic acids were found, namely, chlorogenic acid, p-coumaric acid, ferulic acid, caffeic acid, vanillic acid, and salicylic acid. In addition, stains corresponding to flavonoids were observed in the chromatograms. Analysis of ethanolic bee pollen extracts revealed two phenolic compounds: chlorogenic and gallic acids.

### 3.2. Changes in Bacteria Number Caused by Coated Film Packaging

#### 3.2.1. Comparison of Antibacterial Effectiveness of AP and AS Films during 24 Hours from Contamination

**Figure 1:**
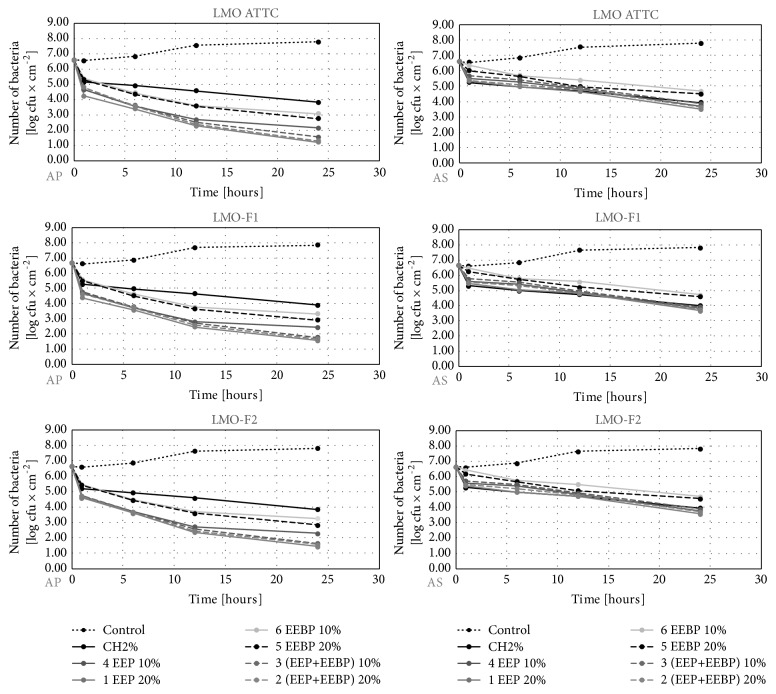
Logarithmic reduction rate of* Listeria monocytogenes* on salmon filet (AP: polypropylene film used directly after preparation, AS: polypropylene film stored for 10 days before usage).

**Figure 2:**
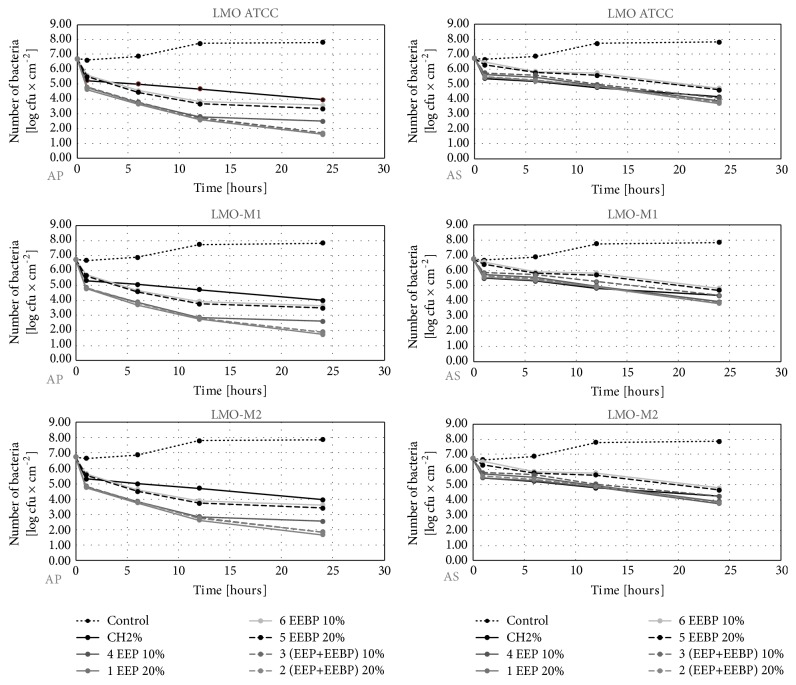
Logarithmic reduction rate of* Listeria monocytogenes* on salami (AP: polypropylene film used directly after preparation, AS: polypropylene film stored for 10 days before usage).

**Figure 3:**
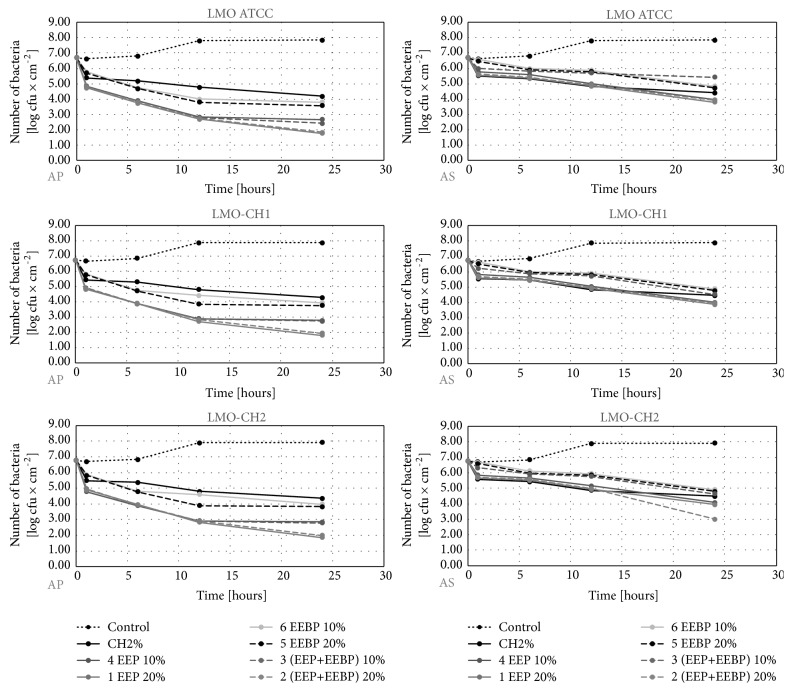
Logarithmic reduction rate of* Listeria monocytogenes* on soft cheese (AP: polypropylene film used directly after preparation, AS: polypropylene film stored for 10 days before usage).

The initial number of* L. monocytogenes* reisolated from the studied slices before their wrapping in the film ranged from 6.6 to 6.8 log CFU×cm^−2^ depending on the type of food and the strain (Figures 1–3). In the control variant (uncoated film), during 24 hours, the number of* L. monocytogenes *counts reached the level of 7.8-7.9 log CFU×cm^−2^ (Figures 1–3). In the experimental variants, we reported a decrease in the number of* L. monocytogenes*. Recovery ranged from 1.2 log CFU×cm^−2^ (the LMO reference strain on salmon filet) to 1.9 log CFU×cm^−2^ (LMO-CH2 strain on cheese) for AP film and from 3.5 log CFU×cm^−2^ (the LMO reference strain on salmon filet) to 4.0 log CFU×cm^−2^ (LMO-CH2 strain on cheese) for the AS film (Figures 1–3). The largest number of* L. monocytogenes* was reisolated after the use of film coated with the solution of chitosan with 10% EEBP. Recovery ranged from 3.1 log CFU×cm^−2^ (the LMO reference strain on salmon filet) to 4.0 log CFU×cm^−2^ (LMO-CH2 strain on cheese) for the AP film and from 4.7 log CFU×cm^−2^ (the LMO reference strain on salmon filet) to 4.9 log CFU×cm^−2^ (LMO-CH2 strain on cheese) for the AS film, respectively (Figures 1–3). The differences in final number observed for* L. monocytogenes *strains isolated from the same kind of food were negligible and not statistically significant (in case of the same coating solution and film type) (Figures 1–3). The results of the study by Torlak and Sert [22] demonstrated that the chitosan-coated film exhibited antibacterial activity against foodborne pathogens, including* L. monocytogenes*. EPP added to coating at 10.0% enhanced antibacterial activity against all tested foodborne pathogens [22].

**Figure 4:**
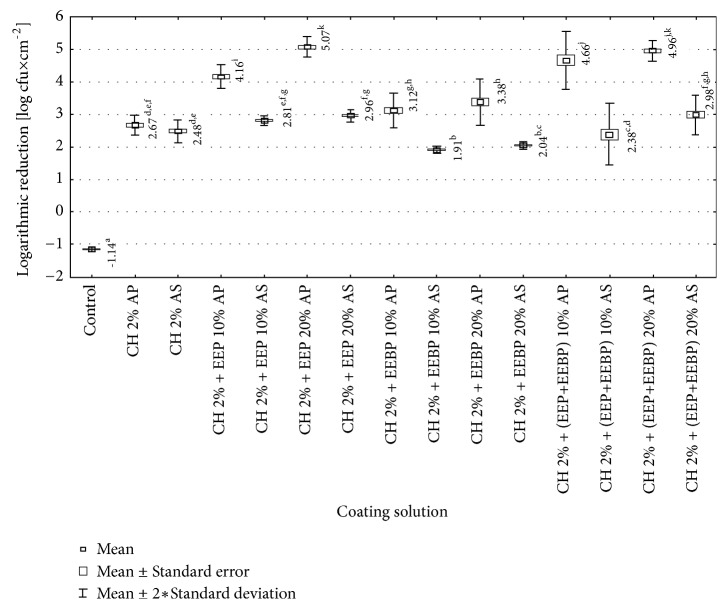
Logarithmic reduction rate of* Listeria monocytogenes* on film used directly after preparation (AP) and after 10-day storage (AS) (a, b, c,…: differences between values marked with different letters are statistically significant).

In our study the chitosan-coated film also significantly reduced number of* L. monocytogenes* counts. The greatest reduction was achieved for AP films though AS films were still active against* L. monocytogenes* strains. The solutions used to coat the films were ranked according to their decreasing biocidal efficacy. For the AP films the order was as follows: chitosan 2% + 20% EEP >chitosan 2% + 20% (EEP+EEBP) >chitosan 2% + 10% (EEP+EEBP) >chitosan 2% + 10% EEP >chitosan 2% + 20% EEBP >chitosan 2% + 10% EEBP> chitosan 2%. Statistically significant differences in effectiveness were found between tested coating solutions, except for these containing chitosan with 10% (EEP+EEBP) and 20% (EEP+EEBP), and chitosan with 10% EEBP and 20% EEBP (Figure 4). For the AS film the effectiveness was as follows: chitosan 2% + 20% (EEP+EEBP) > chitosan 2% + 20% EEP > chitosan 2% + 10% EEP > chitosan 2% >chitosan 2% + 10% (EEP+EEBP) > chitosan 2% + 20% EEBP >chitosan 2% + 10% EEBP. The statistically significant differences in effectiveness were found between chitosan solution + 10% (EEP+EEBP) and chitosan solution + 20% (EEP+EEBP) (Figure 4).

Torlak and Sert [22] reported also that chitosan in the form of film is unable to diffuse through the adjacent media. The high antimicrobial activity of acetic acid chitosan-coated film applied on fresh shredded black radish samples was demonstrated also in Jovanović et al. [17] studies. The initial number of* L. monocytogenes *ATCC 19155 decreased to an undetectable level after three days of refrigerated storage at 4°C. The application of acetic acid chitosan coating solutions in concentrations of 1.0% and 0.5% on black radish samples caused immediate cycle reduction by 3.1 log_10_ CFU/g and 2.6 log_10_ CFU/g, respectively. A higher inhibition of* L. monocytogenes* was achieved at higher chitosan concentration. On the other hand, Siripatrawan and Vitchayakitti [24] demonstrated the lack of antibacterial properties of chitosan film against* Staphylococcus aureus, Salmonella *Enteritidis*, Escherichia coli,* and* Pseudomonas aeruginosa*. Antimicrobial activity was observed only after incorporation of propolis extract into the chitosan film, in concentration ranging between 2.5% and 20.0%. The propolis chitosan film exhibited antimicrobial activity only on the contact surface underneath the film.

Obtained results showed that usage of AP film caused statistically significant greater reduction in number of* L. monocytogenes *strains than usage of AS film, in case of all tested coating solutions, expect 2% chitosan (Figure 4). In each of the studied solutions, the AP and AS film coated with the solution with EEP caused a higher decrease in the number of* L. monocytogenes *than the solutions with EEBP. For the AP film these differences were larger than for AS film, but in both cases they were statistically significant (Figure 4). The explanation of differences in efficiency between EEP and EEBP can be found in Mohdaly et al. [25]. They showed that propolis contains a much higher content of phenolic compounds and thus has a higher antibacterial activity than bee pollen. As main phenolic compounds in the extract of propolis, they detected caffeic acid, ferulic acid, rutin, and p-coumaric acid. In contrast, 3,4-dimethoxycinnamic acid was the main phenolic compound in the bee pollen extract. The stabilization coefficient of propolis extract was 13.7 and pollen 6. They showed that the MIC value for* L. monocytogenes* of bee pollen was 0.30 ± 0.2 mg / ml, and for propolis 0.20 ± 0.02 mg / ml [25]. In turn, results of Mascheroni et al. [26] research may be somewhat useful to explain the differences in antibacterial efficacy between AP and AS films. The researchers have observed that the various components of propolis are characterized by different diffusivity values. The diffusion of ingredients may be a result of contact with food, but it may occur also under the environmental impact of the film, e.g., moisture [26]. This may indicate that the quantitative and qualitative composition of the substances released into the packaged food may be slightly different for AP and AS films.

#### 3.2.2. Effect of Freshly Coated Film on Changes in the Number of L. monocytogenes in Food during Its Storage for 15 Days

**Figure 5:**
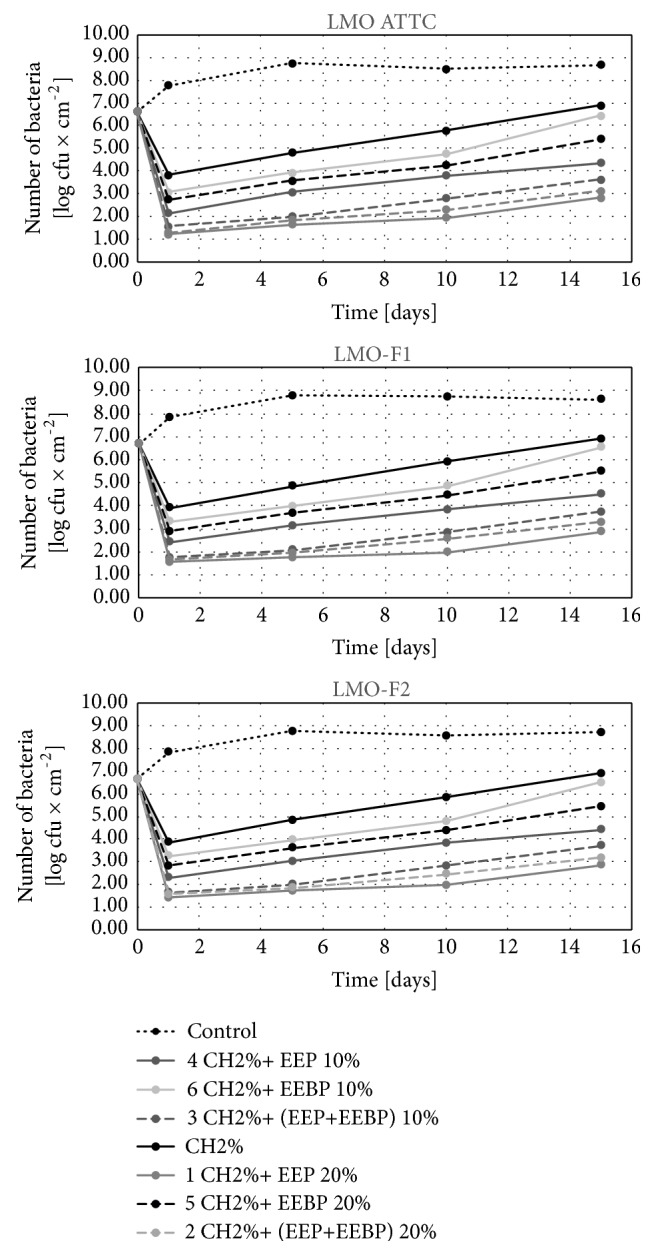
Logarithmic reduction rate of* Listeria monocytogenes* on salmon filet after storage.

**Figure 6:**
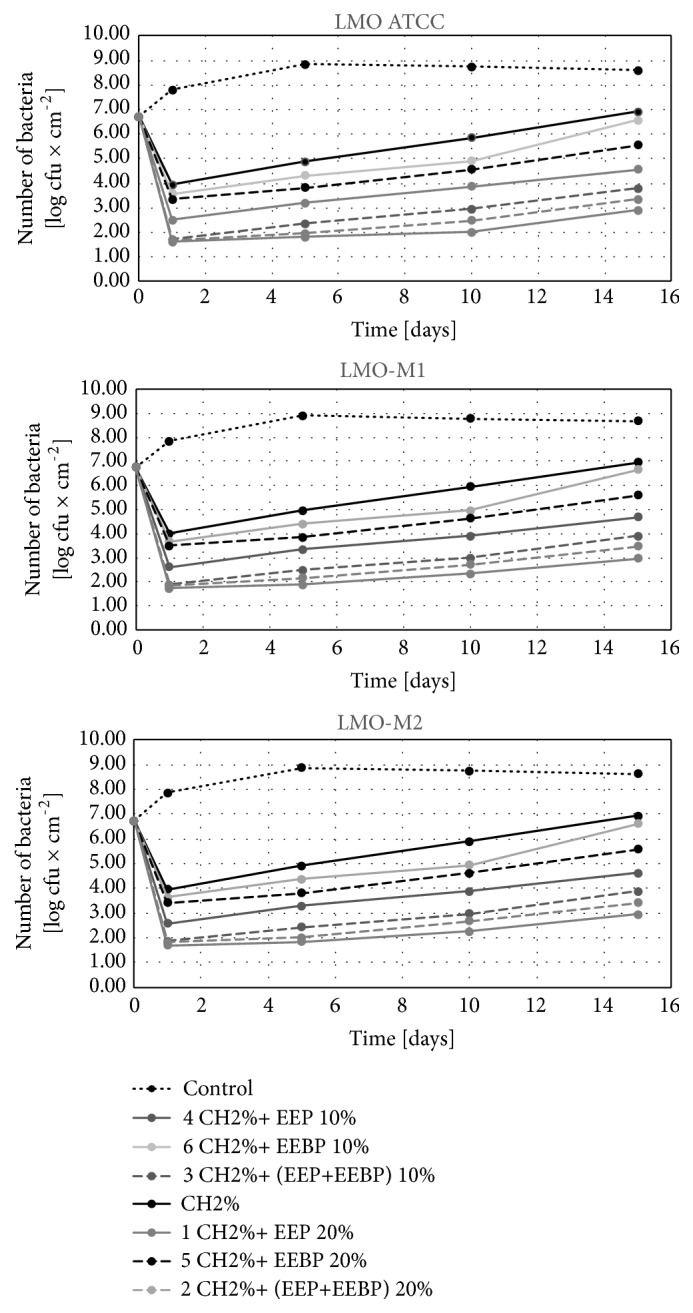
Logarithmic reduction rate of* Listeria monocytogenes* on salami after storage.

**Figure 7:**
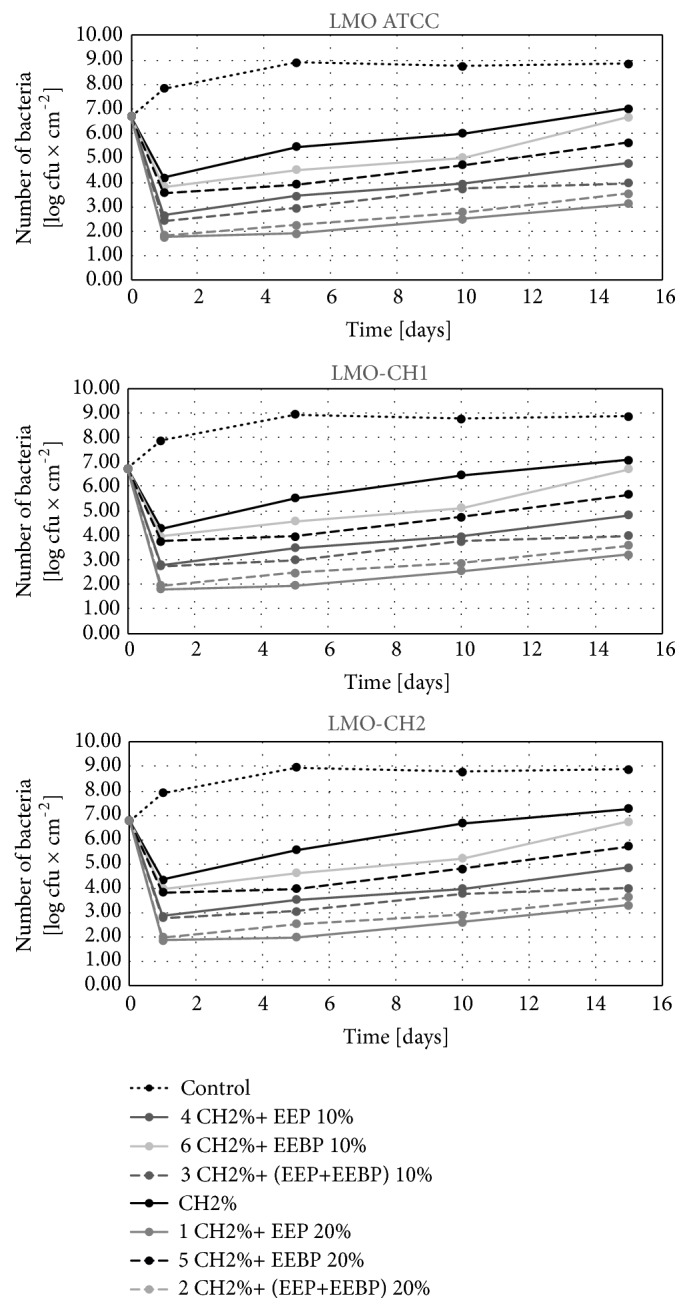
Logarithmic reduction rate of* Listeria monocytogenes* on cheese after storage.

In the control variant, the number of* L. monocytogenes* on the last day of storage stayed at the level from 8.6 log CFU×cm^−2^ (LMO ATCC on salami) to 8.9 log CFU×cm^−2^ (LMO-CH2 on cheese) (Figures 5–7). Among solutions with bee products, the lowest number of these bacteria after 15 days of storage was found for the solution CH 2% + 20% EEP (2.8 log CFU×cm^−2^ for LMO ATCC on salmon filet – 3.3 log CFU×cm^−2^ for LMO-CH2 on cheese), and the highest for the film coated with the solution CH 2% + 10% EEBP (6.5 log CFU×cm^−2^ for LMO ATCC on salmon filet – 6.8 log CFU×cm^−2^ for LMO-CH2 on cheese). For the chitosan solution, the number of* L. monocytogenes* recovered from the stored food ranged from 6.9 log CFU×cm^−2^ for LMO ATCC on salmon filet to 7.3 log CFU×cm^−2^ for LMO-CH2 on cheese (Figures 5–7). The differences in final number observed for* L. monocytogenes *strains isolated from the same kind of food were not statistically significant (in case of the same coating solution and film type) (Figures 5–7). Study of Ye et al. [27] demonstrated that chitosan-coated plastic films are not able to inhibit growth of* L. monocytogenes* on ham steaks. Moreover, the incorporation of antimicrobials (nisin, sodium lactate, sodium diacetate, potassium sorbate, and sodium benzoate) to coating solutions reduced or inhibited the growth of examined bacteria during 10 days of storage. The same group [28] evaluated the effectiveness of chitosan-coated plastic films with incorporated previously described antimicrobials against* L. monocytogenes* on cold-smoked salmon. Chitosan-coated plastic films containing nisin, sodium lactate, and potassium sorbate completely inhibited the growth of* L. monocytogenes *for at least 6 weeks in the refrigerator temperature. The study by Pranoto et al. [29] demonstrated that incorporation of garlic oil into the chitosan film led to an increase in antimicrobial effectiveness against* Escherichia coli, Staphylococcus aureus, Salmonella *Typhimurium*, L. monocytogenes, *and* Bacillus cereus *and did not affect the physical and mechanical properties of chitosan films.

**Figure 8:**
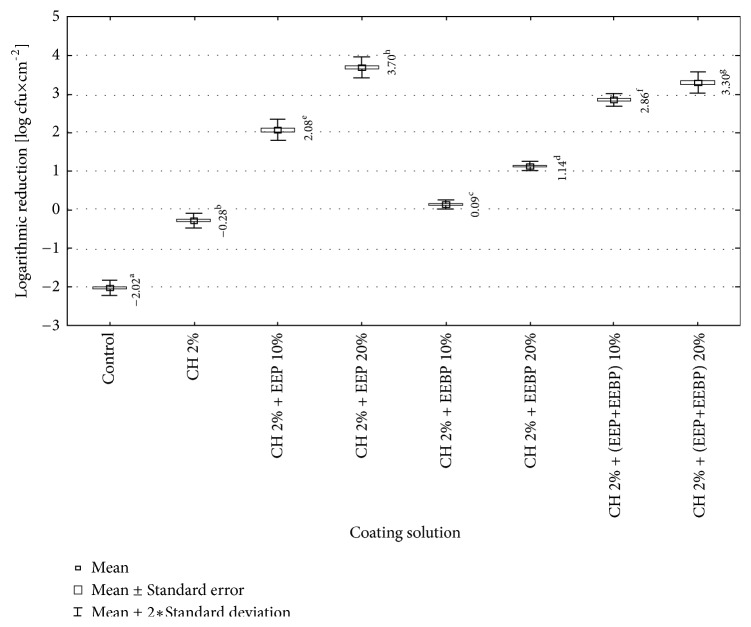
Logarithmic reduction rate of* Listeria monocytogenes *depending on coating solution (a, b, c,…: differences between values marked with different letters are statistically significant).

The analysis of the calculated logarithmic reduction rates revealed that during 15 days of food storage the number of* L. monocytogenes* increased above the initial contamination level in the control variant. In chitosan-coated variants the number of bacteria increased during 15-day storage compared to the initial reduction rate but was still significantly lower than that for the control variant. In the control variant, the values of logarithmic reduction rates stayed on the level of -2.0 log CFU×cm^−2^ and were statistically significantly higher than for the film with chitosan, -0.3 log CFU×cm^−2^ (Figure 8). Similar results were observed for propolis chitosan-coated film by Barrera et al. [30]. They evaluated the effect of 5% EEP containing 1% chitosan film on the antifungal and physicochemical properties of papaya fruits. The fruits covered with the chitosan-EEP film demonstrated a reduction in fungal infection caused by* Colletotrichum gloeosporioides *as compared with the control papaya. In the present study, the EEP solution showed higher effectiveness during storage for 15 days as compared with the EEBP solution.

From the calculated values of logarithmic reduction rates it follows that all the studied coating solutions with added bee products maintained the number of* L. monocytogenes *during food storage at a level below its initial contamination. Their bactericidal effectiveness increased along with the concentration of a bee product in the coating solution and the differences were statistically significant. It was found that the solutions with added EEP provided statistically significantly better food safety than the solutions containing EEBP (Figure 8). De Araújo et al. [31] observed that gelatin films with 40 and 200 g of EEP/100 g of gelatin showed antimicrobial activity against* Staphylococcus aureus *and* Escherichia coli*. The antimicrobial activity was mainly attributed to phenolic compound of EEP and concentration of EEP. Also, Dziedzic et al. [20] showed antibacterial effectiveness of EEP in concentrations ranging from 25 mg/mL to 0.025 mg/mL on mutans* streptococci* group bacteria and lactobacilli saliva residents, while lactobacilli were more susceptible to EEP.

## 4. Conclusions

The present study has proved the bactericidal activity of the film coated with all the tested substances. The effectiveness of the film coated was affected by the composition of the coating solution, the concentration of studied substances, the type of food, and the time that passed from coating of the film and its use. The most effective coating solution against* L. monocytogenes *was that composed of 2% CH and 20% EEP. The propylene film with EEP has potential to be used as packing material against* L. monocytogenes *which will have a broad application in food industry. Moreover, the coated polypropylene films limit the proliferation of* L. monocytogenes* during storage of food, so they can extend shelf-life of packed product. Further studies involving other species of foodborne bacteria are needed.

## Data Availability

The data used to support the findings of this study are included within the article.
